# Inhibition of miR-96-5p alleviates intervertebral disc degeneration by regulating the peroxisome proliferator-activated receptor γ/nuclear factor-kappaB pathway

**DOI:** 10.1186/s13018-023-04412-1

**Published:** 2023-12-01

**Authors:** Xusheng Li, Qian Hou, Wenqi Yuan, Xuehua Zhan, Haifeng Yuan

**Affiliations:** 1 Department of Spine Surgery, General Hospital of Ningxia Medical University, No. 804 Shengli Street, Xingqing District, Yinchuan, 750004 China; 2 Faculty of Medicine, Universiti Teknologi MARA, Sungai Buloh Campus, Jalan Hospital, Sungai Buloh, 47000 Malaysia

**Keywords:** MiR-96-5p, Peroxisome proliferator-activated receptor γ/nuclear factor-kappaB (PPARγ/NF-κB), Intervertebral disc degeneration (IDD), Inflammation, Extracellular matrix (ECM)

## Abstract

**Background:**

Intervertebral disc degeneration (IDD) is the main pathogenesis of low back pain. MicroRNAs (miRNAs) have been found to exert regulatory function in IDD. This study aimed to investigate the effect and potential mechanism of miR-96-5p in IDD.

**Methods:**

In vitro cell model of IDD was established by treating human nucleus pulposus cells (HNPCs) with interleukin-1β (IL-1β). The level of peroxisome proliferator-activated receptor γ (PPARγ) was examined in the IDD cell model by Western blot and quantification real-time reverse transcription-polymerase chain reaction (qRT-PCR). The expression level of miR-96-5p was detected by RT-qPCR. Effects of PPARγ or/and PPARγ agonist on inflammatory factors, extracellular matrix (ECM), apoptosis, and nuclear factor-kappaB (NF-κB) nuclear translocation were examined through enzyme-linked immunosorbent assay (ELISA), Western blot, flow cytometry assay, and immunofluorescence staining. The Starbase database and dual luciferase reporter assay were used to predict and validate the targeting relationship between miR-96-5p and PPARγ, and rescue assay was performed to gain insight into the role of miR-96-5p on IDD through PPARγ/NF-κB signaling.

**Results:**

PPARγ expression reduced with concentration and time under IL-1β stimulation, while miR-96-5p expression showed the reverse trend (*P* < 0.05). Upregulation or/and activation of PPARγ inhibited IL-1β-induced the increase in inflammatory factor levels, apoptosis, degradation of the ECM, and the nuclear translocation of NF-κB (*P* < 0.05). MiR-96-5p was highly expressed but PPARγ was lowly expressed in IDD, while knockdown of PPARγ partially reversed remission of IDD induced by miR-96-5p downregulation (*P* < 0.05). MiR-96-5p promoted NF-κB entry into the nucleus but PPARγ inhibited this process.

**Conclusion:**

Inhibition of miR-96-5p suppressed IDD progression by regulating the PPARγ/NF-κB pathway. MiR-96-5p may be a promising target for IDD treatment clinically.

## Introduction

Lower back pain affects almost one in ten people worldwide and is an important cause of disability [1, 2]. While surgery is currently the most effective therapy, despite treatment, many patients continue to endure chronic pain, sciatica, functional limitations, and a greatly diminished quality of life [3–5]. Intervertebral disc degeneration (IDD) is a predominant cause of low back pain, which places an enormous burden on the individual and society [6, 7]. Intervertebral discs (IVDs) are composed of the nucleus pulposus (NP), the cartilaginous endplates, and the annulus fibrosus [8]. The extracellular matrix (ECM) secreted by the NP maintains homeostasis and is composed of type II collagen (Col II) and proteoglycans, which are substrates for matrix metalloproteinases (MMPs), and are involved in the viscoelastic properties of IVD [9]. During IDD progression, human nucleus pulposus cells (HNPCs) secrete excessive pro-inflammatory molecules such as interleukin-1β (IL-1β) [10, 11]. IL-1β can exacerbate the progression of IDD by catabolizing the activity of metabolic enzymes, including MMPs [10, 12]. It is believed that the pathogenesis of IDD is largely due to the loss of HNPCs in IVD, the alterations in ECM components, and the infiltration of inflammatory factors caused by IL-1β [13]. And IL-1β can also cause the loss of HNPCs by inducing apoptosis [14, 15]. However, present therapy for IDD is centered on relieving symptoms rather than addressing the cause of degeneration [16], and deeper excavation of the biomolecular mechanisms of IDD progression is imminent for further treatment of IDD.

Peroxisome proliferator-activated receptors (PPARs) include three subtypes, namely PPARα, PPARγ, and PPARδ, all of which are nuclear receptors [17]. Besides its well-known regulatory role in lipid and glucose metabolism, PPARγ can repress the inflammatory process [18, 19], and previous studies have shown its activation suppressed IL-1β-induced inflammation in human osteoarthritis chondrocytes. This suggests PPARγ may be an attractive therapeutic target for osteoarthritis [20, 21]. Moreover, activated PPARγ plays an anti-inflammatory role in protecting the cardiovascular system by inhibiting nuclear factor-kappaB (NF-κB)-induced cytokines [22]. Ge et al*.* showed chlorogenic acid improved IDD by inhibiting NF-κB signaling [23], and Liu et al*.* reported andrographolide suppressed the degeneration of HNPCs via repressing the NF-κB pathway [24]. Further, Zhang et al*.* found the NF-κB pathway was suppressed by miR-150 in IDD [25]. It is also found that activating PPARγ can alleviate IDD inflammation by inhibiting NF-κB [26]. Based on these reports, it is suggested that NF-κB is regulated by PPARγ, and both may be involved in the regulation of apoptosis and inflammation in IDD.

MicroRNAs (miRNAs) are small, noncoding, endogenous, and highly conserved RNAs with a length of ~ 21–25 bases [27–29], and several reports have indicated they influence the occurrence and development of orthopaedic diseases in multiple ways, including osteoarthritis [30], tendon injuries [31], and IDD [32, 33]. The level of miR-486-5p in lipopolysaccharide (LPS)-induced HNPCs showed a significant downward trend and could inhibit LPS-induced apoptosis and hinder the secretion of matrix-degrading enzymes and inflammatory cytokines [32]. Similarly, miR-154 was upregulated in HNPCs, and its suppression promoted the level of ECM and decreased the level of matrix degrading enzymes [33]. Based on the Starbase prediction results, PPARγ was found to have a target binding site of miR-96-5p, which has been described as having an important role in various orthopedic diseases. For example, Ormseth et al*.* showed miR-96-5p could be used as a biomarker for the treatment of arthritis [34], and Yang et al*.* showed it targeted factor receptor substrate 2 (FRS2) to promote apoptosis in nuclear myeloid cells [35]. In addition, lncRNA LNC_000052 caused the malfunction of mesenchymal stem cells in osteoporosis via the miR-96-5p-phosphoinositide-3-kinase regulatory subunit 1 (PIK3R1) axis [36]. These reports indicate that miR-96-5p, which can target PPARγ, is involved in the regulation of inflammation and apoptosis, which is also the mechanism of IDD, suggesting that miR-96-5p is involved in the progression of IDD.

Based on the above literature reports and predictions of targeting relationships, it is speculated that miR-96-5p regulates NF-κB by targeting the expression of PPARγ, thereby participating in the occurrence and progression of IDD. This study mainly analyzes the targeting effect of miR-96-5p on PPARγ and the regulatory mechanism of NF-κB, as well as the impact of this regulatory axis on apoptosis and inflammation of HNPCs, thereby analyzing its role in IDD.

## Methods

### Human tissue samples

Three patients with severe IDD (average age: 41.5 ± 6.1 years) (IDD grade IV) and three patients with mild IDD (average age: 40.2 ± 2.9 years) (IDD grade I) were operated on and tissue specimens were collected. The degree of IDD progression was determined by magnetic resonance imaging (MRI) and assessed according to the Pfirrmann classification. Patient specimens were obtained from patients with surgically resected herniated discs and IDD, and surgical specimens were transported to the laboratory for sterile culture. All granulation tissue in the sample was carefully removed, leaving only the disc tissue, before tissue specimens were placed in a – 80 °C refrigerator for subsequent experiments. The study conformed to the provisions of the Declaration of Helsinki (as revised in 2013), and was performed with the approval of the Medical Ethics Committee of General Hospital of Ningxia Medical University (No. 2019–31), and all participants signed the written informed consent.

### Cell culture

HNPCs isolated from the NP of human IVDs were purchased from ScienCell Research Laboratories. Cells were cultured in the Nucleus Pulposus Cell Medium (NPCM, Cat. #4801) in an incubator at 37 °C and 5% CO_2_. In order to find the optimal experimental concentration, IL-1β at final concentrations of 0, 0.5, 1.0, 5.0 and 10.0 ng/mL were added to the culture medium and incubated for 48 h. And HNPCs were cultured at 10 mg/mL IL-1β for 0, 6, 12, 24 and 48 h. PPARγ was activated by incubating HNPCs with 1 μM of the PPARγ agonist pioglitazone for 24 h.

### Cell transfection

HNPCs were divided into eight groups: a normal control (NC) group (without treatment), IL-1β group (treated with IL-1β), IL-1β + PPARγ group (treated with PPARγ and IL-1β), IL-1β + pioglitazone group (treated with pioglitazone and IL-1β), IL-1β + PPARγ + pioglitazone group (treated with PPARγ, IL-1β and pioglitazone), IL-1β + miR-96-5p inhibitor group (treated with miR-96-5p inhibitor and IL-1β), IL-1β + small interfering PPAR (siPPAR) group (treated with siPPAR and IL-1β treatment), and an IL-1β + miR-96-5p inhibitor + siPPAR group (treated with miR-96-5p inhibitor, siPPAR, and IL-1β). The sequence of miR-96-5p inhibitor/NC and PPARγ siRNA was designed and synthesized by RiboBio (Guangzhou, China). After 48 h of cell transfection in lipofectamine 2000 (Invitrogen, Waltham, MA, USA), the transfected cells were further stimulated by medium supplemented with IL-1β for 24 h. SiRNA target sequences of siPPAR: 5′-GTTCAAACACATCACCCCC-3′; inhibitor of miR-96-5p: 5′-GCAAAAATGTGCTAGTGCCAA-3′; NC inhibitor of miR-96-5p: 5′-TAACACGTCTATACGCCCA-3′. The specific transfection method is as follows: 500 μl culture medium and 1 × 10^5^ cells were added to each well of a 24-well culture dish. Diluted plasmid (0.8 μg) was added to 50 μl serum-free medium in a 1.5 ml centrifuge tube, mix gently as A. In another 1.5 ml centrifuge tube, lipofectamine 2000 (2 μl) was dissolved in 50 μl of serum-free medium and mix well. It was incubated at 25 °C for 5 min, mixed gently with A, and incubated at 25 °C for 20 min. 100 μl of the above mixture was added to the cells and mixed gently, and then incubated for 6 h in a 37 °C, 5% CO_2_ incubator. Then replaced the incomplete medium containing the transfection complex with fresh serum-containing medium and continue culturing for 48 h.

### Immunofluorescence staining

HNPCs were seeded into a 24-well plate preplaced with a cover slip then fixed with paraformaldehyde (4%) and permeabilized with Triton X-100 (0.1%) for 10 min, respectively. Goat serum was then adopted to block cells for 30 min. Cells were incubated with 200 μL of anti-NF-κB primary antibody (ab283716, 2 μg/mL, Abcam, Cambridge, MA) for 1 h at 4 °C, then incubated with goat anti-rabbit immunoglobulin G (IgG) (1:1000, ab150077) for 30 min at 25 °C in the dark. Nuclei were stained by 200 μL of 0.1% 4′, 6′-diamidino-2-phenylindole (DAPI), and anti-fluorescence quenching agent was then dropped onto the slide. Laser confocal microscopes (Nikon Instruments Inc., Melville, NY, USA) were used to observe cells and obtain images.

### Dual luciferase reporter gene assay

Two segments from the 3′-untranslated region (3′-UTR) of PPARγ were amplified via PCR and constructed into a luciferase vector (Promega, Madison, WI, USA), then labeled as PPARγ-wild-type (PPARγ-WT). The PPARγ-mutant (PPARγ-MUT) plasmid was obtained by replacing the corresponding vector with a mutated miR-96-5p binding sequence. The PPARγ-WT (or PPARγ-MUT) plasmid conjugate was then transfected with miR-96-5p mimic (5′-UUUGGCACUAGCACAUUUUUGCU-3′) or NC mimic (5′-UCACCGGGUGUAAAUCAGCUUG-3′) into HNPCs for 48 h, and a Dual-Luciferase® Reporter Assay System (Promega) was utilized to determine luciferase activity.

### Quantification real-time reverse transcription-polymerase chain reaction (qRT-PCR)

For the examination of PPARγ messenger RNA (mRNA) and miR-96-5p expression, total RNAs were isolated from HNPCs and NP tissues using TRIzol reagent (Ambion, Austin, TX, USA). Total RNAs were then synthesized into complementary DNA (cDNA) with a TaqMan miRNA Reverse Transcription Kit (Thermo Fisher Scientific, Waltham, MA, USA). QRT-PCR was conducted with SYBR green detection (Qiagen, Valencia, CA, USA), with β-actin and U6 utilized as a control. Each qRT-PCR experiment was conducted at least three times, and the sequence used was as follows: PPARγ: 5′-CATCCTCCCACCCAAICATC-3′ (F) and 5′-GGACCICCAGCAAACACCAG-3′ (R); miR-96-5p: 5′-AGAAGAGAAATCCATGGAGC-3′ (F) and 5′-CICCAACTGTGAAGATCCAGTA-3′ (R); β-actin: 5′- AGACCTGTACGCCAACACAG-3′ (F) and 5′-TTCTGCATCCTGTCGGCAAT-3′ (R); U6: 5′-ATTGGAACGATACAGAGAAGATT-3′ (F): and 5′-GGAACGCTTCACGAATTTG-3′ (R). The 2^−ΔΔCt^ method was adopted to analyze the relative expression of PPARγ mRNA and miR-96-5p.

### Western blot

Cells were lysed with lysis buffer, and proteins in cytoplasm and nucleus were separated by professional Ambion PARISTM kit (Invitrogen, USA). The bicinchoninic acid (BCA) assay was performed to measure protein concentration by the BCA Protein Assay Kit (Pierce, Thermo Fisher Scientific, USA), and sodium dodecyl sulfate–polyacrylamide gel electrophoresis (SDS-PAGE) was performed to separate proteins. Proteins were subsequently transferred to polyvinylidene fluoride (PVDF) membranes, followed by blocking with 5% skim milk (25 °C, 1 h). After washing, the membrane was incubated with the corresponding primary antibodies overnight at 4 °C. The primary antibodies were as follows: anti-PPARγ (ab178860, 1:1,000, Abcam), anti-Col II (ab188570, 1:1,000, Abcam), anti-aggrecan (ab3778; 1:200, Abcam), anti-MMP3 (ab52915, 1:1,000, Abcam), anti-MMP13 (ab51072, 1:1,000, Abcam), anti-A disintegrin and metalloproteinase with thrombospondin motifs type 4 (ADAMTS-4) (ab185722, 1:1,000, Abcam), anti-ADAMTS-5 (ab41037, 1:250, Abcam), anti-NF-κB (ab32360, 1:1,000, Abcam), anti-β-actin (ab8226, 1:1,000, Abcam), and anti-H3 (ab1791, 1:200, Abcam). β-actin was used as internal control for cytoplasmic protein and H3 was used as internal control for cytonuclear protein. The primary antibody on the membrane was conjugated with horseradish peroxidase-labeled secondary antibody (ab6721, 1:2,000, Abcam), and enhanced chemiluminescence was used to develop the protein blots. Image J software [version 1.46r, ImageJ, National Institutes of Health (NIH), Bethesda, MD, USA] was applied for the qualification of proteins.

### Enzyme-linked immunosorbent assay (ELISA)

ECM-related proteins in the culture supernatant of the HNPCs were determined using commercial human-specific ELISA kits (Abcam). The culture supernatant was acquired from the HNPCs treated for 48 h, and the level of the ECM-related proteins was detected at 450 nm.

### Statistical analysis

SPSS 19.0 software (IBM, Armonk, NY, USA) was utilized to analyze the data, which are expressed as mean ± standard deviation. Differences between two groups were contrasted by Student’s *t*-test, and those in at least three groups were determined by one-way analysis of variance with Tukey’s post hoc test. If the P value was less than 0.05, the difference was regarded as significant. All experiments were repeated three times.

## Results

### Decreased PPARγ expression in an IDD cell model induced by IL-1β

**Fig. 1:**
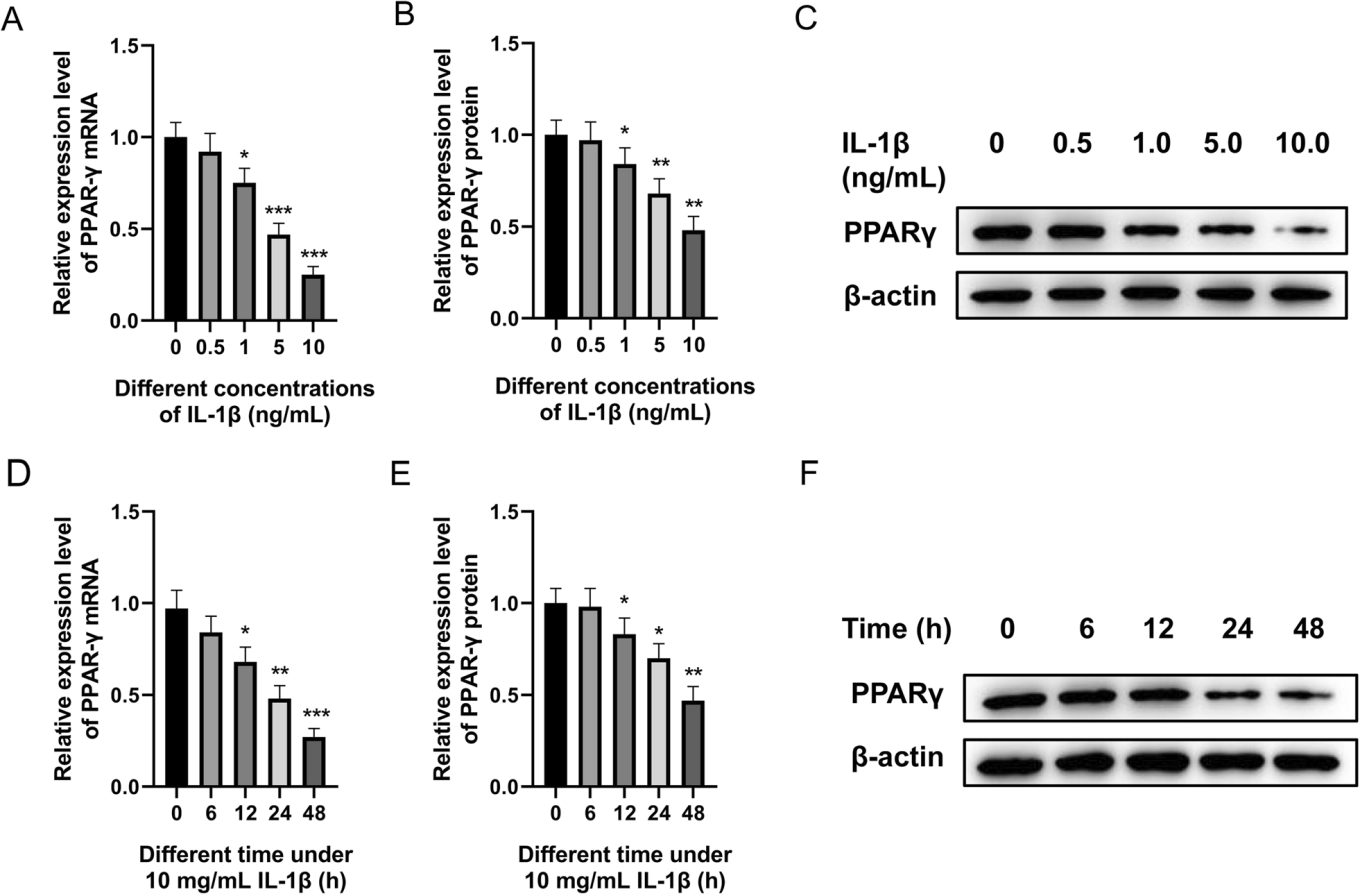
Decreased PPARγ expression in an IDD cell model induced by IL-1β. **A** qRT-PCR was used to detect the expression of PPARγ mRNA in HNPCs cultured with different concentrations of IL-1β for 48 h. **B**, **C** PPARγ protein level detected by Western blot after HNPCs were incubated in different concentrations of IL-1β for 48 h. **D** qRT-PCR was used to detect the expression of PPARγ mRNA after HNPCs were incubated at 10 mg/mL IL-1β for different durations. **E**, **F** PPARγ protein level detected by Western blot after HNPC were incubated at 10 mg/mL IL-1β for different durations. (*P < 0.05, **P < 0.01, ***P < 0.001 *vs.* previous nearby concentration group/time point group). PPARγ, peroxisome proliferator-activated receptor γ; mRNA, messenger RNA; IL-1β, interleukin-1β; IDD, intervertebral disc degeneration; qRT-PCR, quantification real-time reverse transcription-polymerase chain reaction; HNPCs, human nucleus pulposus cells

This research first determined the influence of IL-1β treatment on PPARγ expression, and the results indicated an increase of IL-1β concentration was associated with a steady downward trend in the mRNA level of PPARγ (Fig. 1A, *P* < 0.05, *P* < 0.001), and the protein level of PPARγ detected by Western blot had a similar trend (Fig. 1B, C, *P* < 0.05, *P* < 0.01). In addition, a progressive reduction of PPARγ expression at both mRNA and protein levels was found with the increase of time in HNPCs simulated by 10 mg/mL IL-1β (Fig. 1D-F, *P* < 0.05, *P* < 0.01, *P* < 0.001). These results illustrated the expression of PPARγ gradually decreased with an increasing treatment concentration and exposure time to IL-1β, suggesting PPARγ might be involved in the process of IDD.

### Effect of PPARγ protein upregulation and PPARγ activation on the IDD cell model

**Fig. 2:**
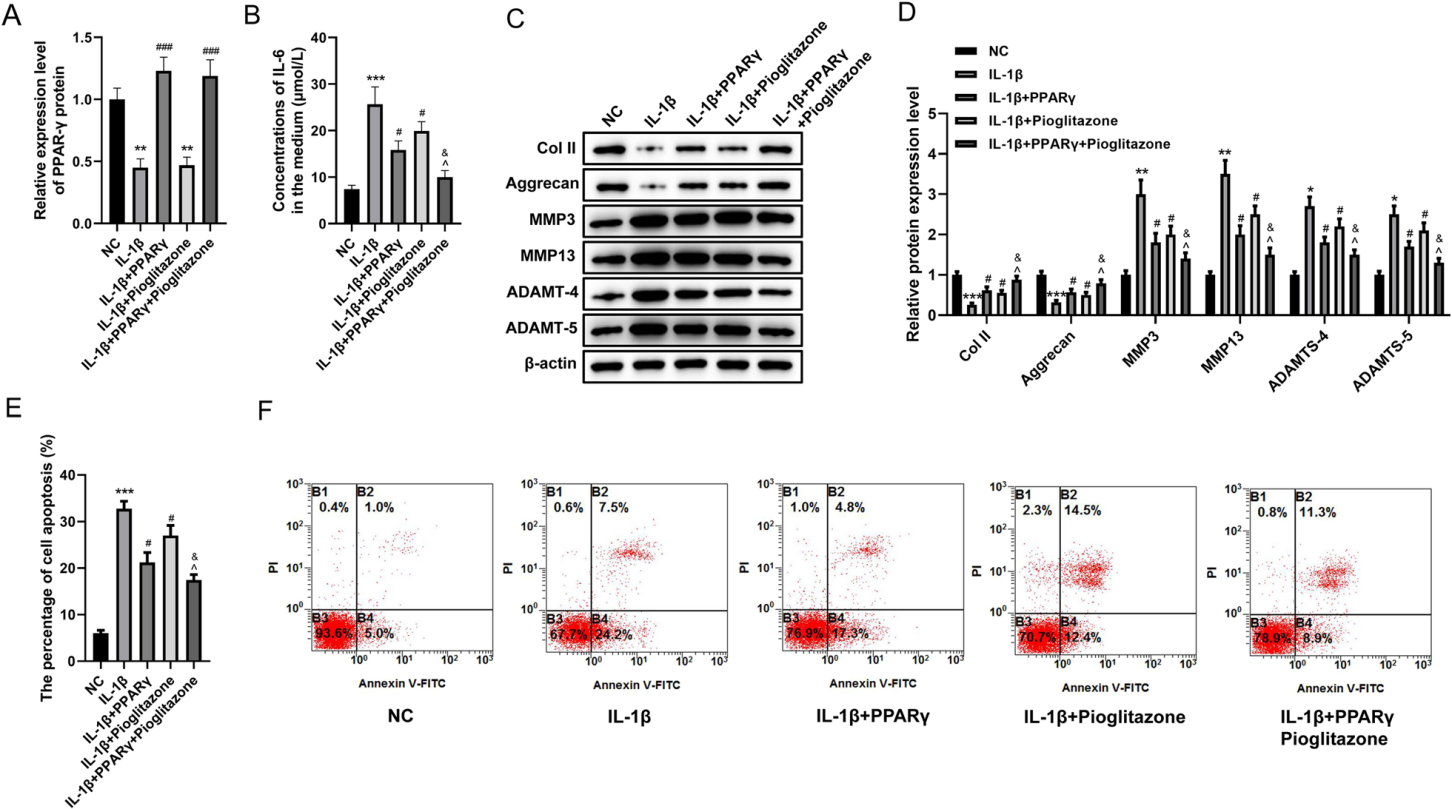
Effect of PPARγ protein upregulation and PPARγ activation on an IDD cell model. HNPCs were treated with IL-1β (and pioglitazone) and /or transfected plasmid PPARγ (groups: NC, IL-1β, IL-1β + PPARγ, IL-1β + pioglitazone, IL-1β + PPARγ + pioglitazone). **A** The protein level of PPARγ was detected by Western blot. **B** Concentration of IL-6 in culture medium was detected by ELISA assay. **C**, **D** Protein expression of Col II, aggrecan, MMP3, MMP13, ADAMT-4, and ADAMT-5 in the ECM was detected by Western blot. **E**, **F** Flow cytometry analysis with Annexin V-PI staining was performed to evaluate the percentage of apoptotic cells in HNPCs. (*P < 0.05, **P < 0.01, ***P < 0.001 *vs.* NC group; ^#^*P* < 0.05, ^###^*P* < 0.001 *vs.* IL-1β group; ^^^*P* < 0.05 *vs.* IL-1β + PPARγ group; ^&^*P* < 0.05 *vs.* IL-1β + pioglitazone group). PPARγ, peroxisome proliferator-activated receptor γ; NC, normal control; IL, interleukin; Col II, type II collagen; MMP, matrix metalloproteinase; ADAMT, A disintegrin and metalloproteinase with thrombospondin motifs; PI, propidium iodide; FITC, fluorescein isothiocyanate; IDD, intervertebral disc degeneration; HNPCs, human nucleus pulposus cells; ELISA, enzyme-linked immunosorbent assay; ECM, extracellular matrix

To investigate whether PPARγ influenced IDD, we explored the effect of its upregulation and activation on an IDD cell model. As shown in Fig. 2A, the PPARγ level decreased in the model and increased after overexpression (*P* < 0.01, *P* < 0.001). The PPARγ agonist pioglitazone had essentially no effect on PPARγ protein expression, and the combination of PPARγ and pioglitazone produced similar results to those of PPARγ alone (Fig. 2A, *P* < 0.001). ELISA assay revealed the concentration of IL-6 in the culture medium supernatant was elevated in the IDD cell model when contrasted with the NC group (*P* < 0.001), while both PPARγ and pioglitazone treatment both alone and in combination significantly decreased the IL-6 level (Fig. 2B, *P* < 0.05). As shown in Fig. 2C, D, an obvious decrease of Col II and aggrecan expression was found in the IDD cell model (*P* < 0.001), while PPARγ significantly increased Col II and aggrecan expression (*P* < 0.05), and pioglitazone further enhanced the effect of PPARγ (*P* < 0.05). However, MMP3, MMP13, ADAMT-4, and ADAMT-5 showed the opposite result (*P* < 0.05). In the IDD cell model, the apoptosis percentage of HNPCs was obviously increased (*P* < 0.001), both PPARγ and pioglitazone reduced the apoptosis percentage of HNPCs (*P* < 0.05), and pioglitazone increased the reduction of the apoptosis rate of HNPCs induced by PPARγ (Fig. 2E, F, *P* < 0.05). Therefore, upregulation of PPARγ protein and activation of PPARγ could reduce the inflammatory response, the degradation of ECM, and apoptosis of HNPCs in an IDD cell model.

### Effect of PPARγ protein upregulation and PPARγ activation on NF-κB in an IDD cell model

**Fig. 3:**
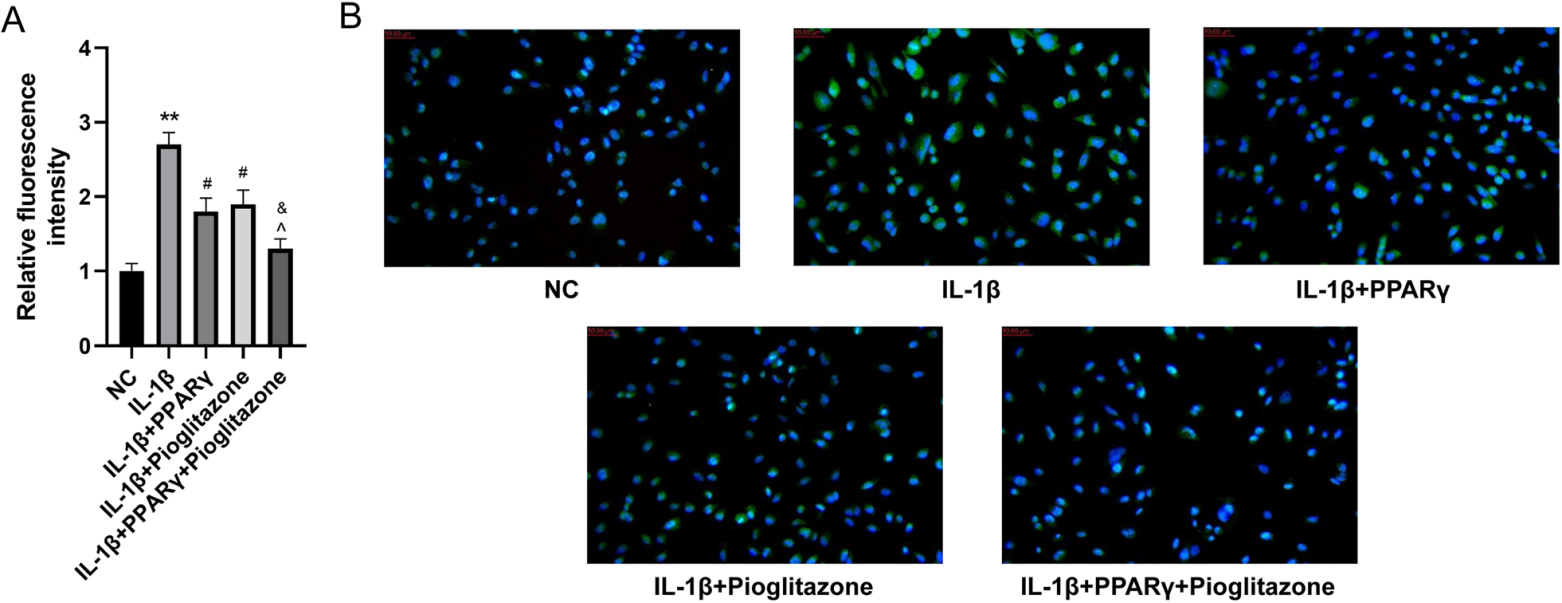
Effect of upregulation and activation of PPARγ protein on NF-κB in an IDD cell model. **A** Histogram showing the fluorescence staining intensity of NF-κB in each group of HNPCs. **B** Immunofluorescence staining of the cell nucleus (blue) and NF-κB (green) in each group of HNPCs. (**P < 0.01 *vs.* NC group; ^#^*P* < 0.05 *vs.* IL-1β group; ^*P* < 0.05 vs. IL-1β + PPARγ group; ^&^*P* < 0.05 *vs.* IL-1β + pioglitazone group). NC, normal control; IL-1β, interleukin-1β; PPARγ, peroxisome proliferator-activated receptor γ; NF-κB, nuclear factor-kappaB; IDD, intervertebral disc degeneration; HNPCs, human nucleus pulposus cells

The influence of PPARγ on NF-κB signaling in the IDD cell model was examined, and the results showed IL-1β stimulation promoted cytoplasmic NF-κB translocation into the nucleus (*P* < 0.01). PPARγ or/and pioglitazone reduced the nuclear translocation of NF-κB after 48 h of IL-1β stimulation when compared with the IL-1β group (*P* < 0.05), while pioglitazone exacerbated the inhibitory effect of PPARγ on NF-κB nuclear translocation (Fig. 3A, B, *P* < 0.05). These results suggested the upregulation and activation of PPARγ protein suppressed the entry of NF-κB into the nucleus triggered by IL-1β.

### PPARγ was targeted by miR-96-5p in HNPCs

**Fig. 4:**
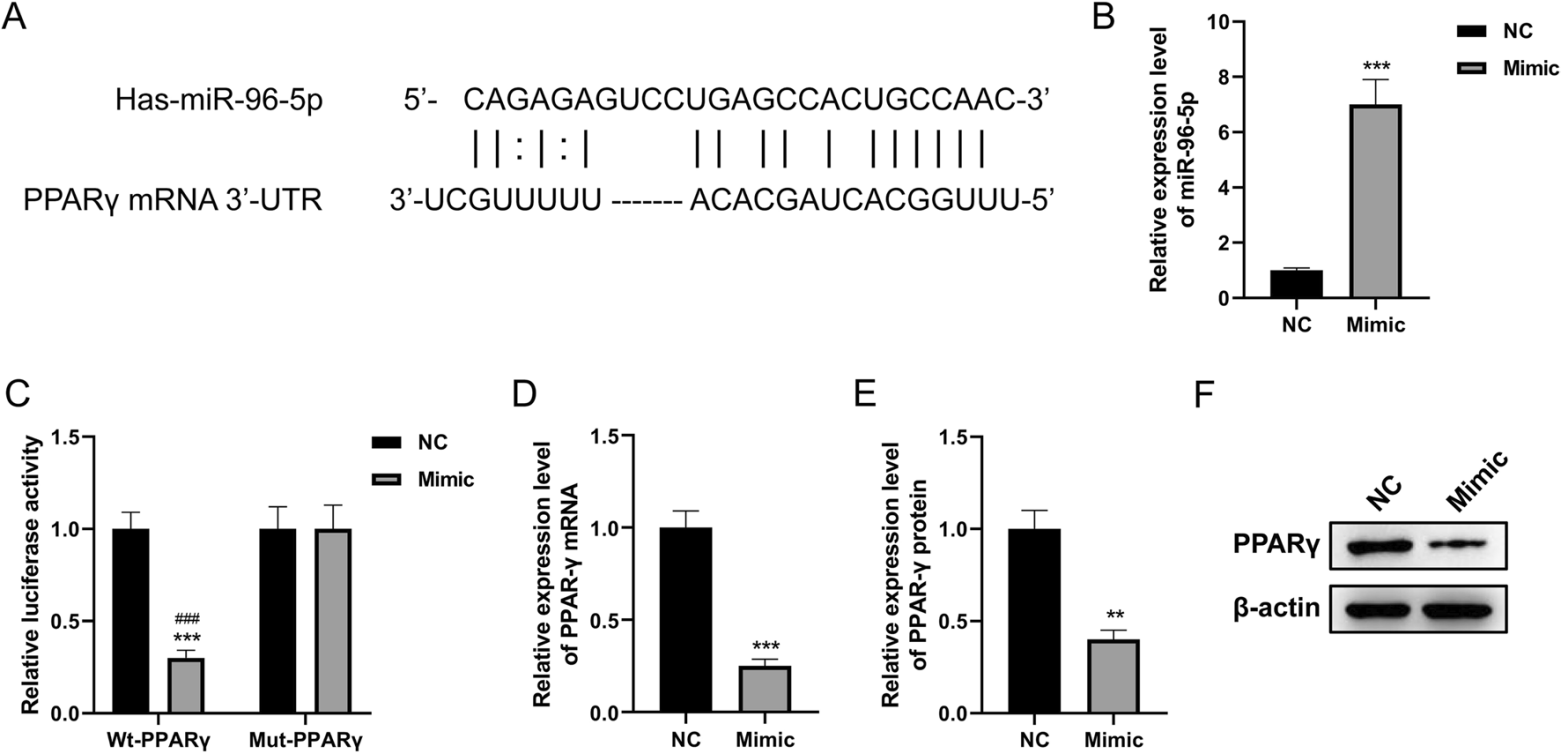
PPARγ was targeted by miR-96-5p in HNPCs. **A** Complementary sites for miR-96-5p and PPARγ as predicted by Starbase database. **B** Analysis of the luciferase activity of the luciferase reporter plasmid containing either WT or MUT PPARγ 3'-UTR in HNPCs following co-transfection with the constructed plasmids PPARγ and miR-96-5p mimics or negative control by luciferase reporter assay. **C** The targeting relationship between miR-96-5p and PPARγ in HNPCs was validated by dual luciferase reporter assay. **D** PPARγ mRNA expression in HNPCs transfected with miR-96-5p mimic detected by qRT-PCR. **E**, **F** PPARγ protein expression in HNPCs transfected with miR-96-5p mimic detected by Western blot. (**P < 0.01, ***P < 0.001 *vs.* NC group; ^###^*P* < 0.001 *vs.* MUT-PPARγ group). PPARγ, peroxisome proliferator-activated receptor γ; mRNA, messenger RNA; 3'-UTR, 3'-untranslated region; NC, normal control; WT, wild-type; MUT, mutant; HNPCs, human nucleus pulposus cells; qRT-PCR, quantification real-time reverse transcription-polymerase chain reaction

To analyze the underlying molecular mechanism of PPARγ regulating IDD, bioinformatics analysis was conducted using Starbase (https://starbase.sysu.edu.cn/) to find the target miRNA of PPARγ, and the results showed miR-96-5p contains a sequence complementary to PPARγ (Fig. 4A). To investigate the possibility of miR-96-5p directly target PPARγ, we then successfully overexpressed miR-96-5p (Fig. 4B, *P* < 0.001) and performed a dual luciferase reporter gene assay, which revealed miR-96-5p mimic significantly decreased the luciferase activity of WT-PPARγ but not that of MUT-PPARγ (Fig. 4C, *P* < 0.001). Further, the mRNA and protein levels of PPARγ were remarkably declined in the miR-96-5p overexpressed HNPCs (Fig. 4D-F, *P* < 0.01, *P* < 0.001). These results indicated miR-96-5p directly targeted PPARγ.

### MiR-96-5p was upregulated in IDD

**Fig. 5:**
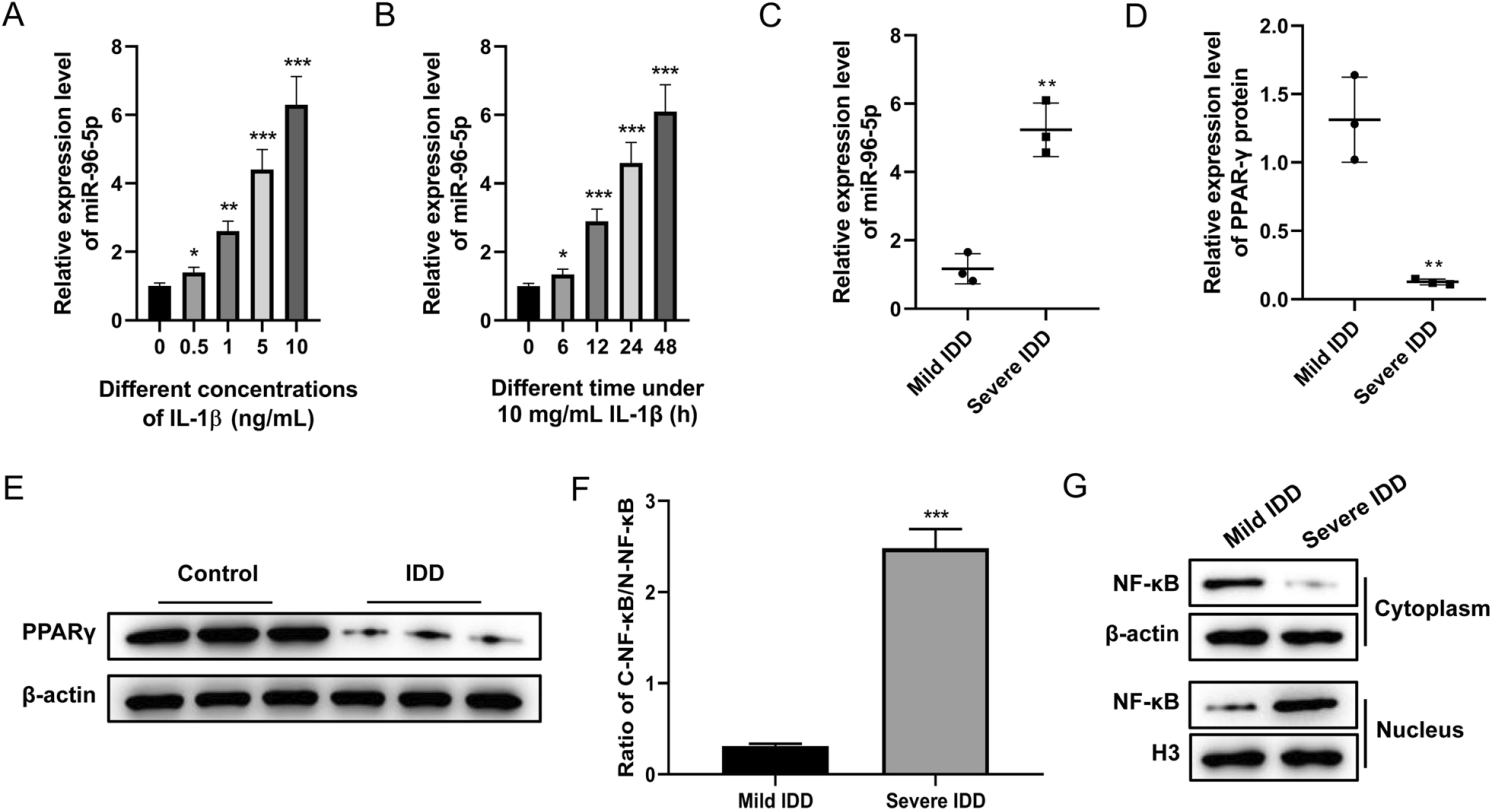
Expression characteristics of miR-96-5p in IDD. **A** qRT-PCR revealed the concentration of miR-96-5p in HNPCs after incubation with indicated concentrations of IL-1β for 48 h. **B** qRT-PCR was used to confirm the value of miR-96-5p in HNPCs after incubation with 10 mg/mL concentration of IL-1β for various times. **C** The expression of miR-96-5p was assessed by qRT-PCR in degenerated IVD NP tissues of a mild IDD group and severe IDD group. **D**, **E** Western blot assay was used to analyze the protein expression of PPARγ in degenerated IVD NP tissues of a mild IDD group and severe IDD group. **F**, **G** Western blot assay was used to analyze the protein expression of NF-κB in the cytoplasm and nucleus of degenerated IVD NP tissues in a mild IDD group and a severe IDD group. (*P < 0.05, **P < 0.01, ***P < 0.001 *vs.* previous nearby concentration group/time point group/Mild IDD group). IL-1β, interleukin-1β; IDD, intervertebral disc degeneration; PPARγ, peroxisome proliferator-activated receptor γ; NF-κB, nuclear factor-kappaB; qRT-PCR, quantification real-time reverse transcription-polymerase chain reaction; HNPCs, human nucleus pulposus cells; IVD, intervertebral disc; NP, nucleus pulposus

The level of miR-96-5p in IDD was detected, and Fig. 5A showed this increased under IL-1β treatment by means of concentration dependence (*P* < 0.05, *P* < 0.01, *P* < 0.001) and when the duration of IL-1β stimulation was gradually prolonged (Fig. 5B, *P* < 0.05, *P* < 0.001). In addition, miR-96-5p was upregulated (Fig. 5C, *P* < 0.01) but PPARγ was significantly downregulated in severe IDD tissue compared to the mild group (Fig. 5D, E, *P* < 0.01). In contrast to the mild, a much higher expression of NF-κB was found in the nuclear extract prepared from the severe IDD tissues (Fig. 5F, G, *P* < 0.001). These data suggested miR-96-5p, PPARγ, and NF-κB might be associated with IDD progression.

### Inhibition of miR-96-5p relieved IDD in HNPCs by regulating PPARγ

**Fig. 6:**
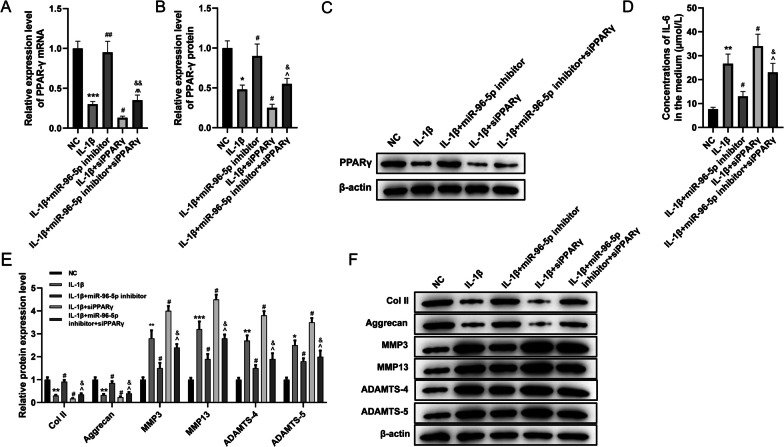
Inhibition of miR-96-5p reversed IL-1β-induced inflammatory factors and ECM changes in HNPCs by regulating the level of PPARγ. HNPCs were treated with IL-1β and /or transfected with miR-96-5p inhibitor and/or short hairpin PPARγ (groups: NC, IL-1β, IL-1β + miR-96-5p inhibitor, IL-1β + siPPARγ, IL-1β + miR-96-5p inhibitor + siPPARγ). **A** The mRNA level of PPARγ was determined by qRT-PCR. **B**, **C** The protein expression of PPARγ in HNPCs of each group was detected by Western blot. **D** The concentration of IL-6 in culture medium was detected by ELISA assay. **E**, **F** The protein expression of Col II, aggrecan, MMP3, MMP13, ADAMTS-4, and ADAMTS-5 related to ECM were detected by Western blot. (*P < 0.05, **P < 0.01, ***P < 0.001 *vs.* NC group; ^#^*P* < 0.05, ^##^*P* < 0.01 *vs.* IL-1β group; ^^^*P* < 0.05, ^^^^*P* < 0.01 *vs.* IL-1β + miR-96-5p inhibitor; ^&^*P* < 0.05, ^&&^*P* < 0.01 *vs.* IL-1β + siPPARγ). PPARγ, peroxisome proliferator-activated receptor γ; mRNA, messenger RNA; IL, interleukin; NC, normal control; siPPARγ, small interfering PPARγ; Col II, type II collagen; MMP, matrix metalloproteinase; ADAMT, A disintegrin and metalloproteinase with thrombospondin motifs; ECM, extracellular matrix; HNPCs, human nucleus pulposus cells; qRT-PCR, quantification real-time reverse transcription-polymerase chain reaction; ELISA, enzyme-linked immunosorbent assay

To further explore whether miR-96-5p regulates IDD through PPARγ, a rescue experiment was performed. This revealed a miR-96-5p inhibitor caused a significant increase of PPARγ expression at mRNA and protein levels compared with the IDD cell model (*P* < 0.05, *P* < 0.01), but knockdown of PPARγ partially reversed this (Fig. 6A-C, *P* < 0.05, *P* < 0.05). In addition, inhibition of miR-96-5p resulted in a reduction in IL-6 level in the medium (*P* < 0.05), and knockdown of PPARγ partially restored this decrease (Fig. 6D, *P* < 0.05). MiR-96-5p increased Col II and aggrecan expression and decreased MMP3, MMP13, ADAMTS-4, and ADAMTS-5 levels in the IDD cell model (*P* < 0.05). Intriguingly, this trend was partially reversed by siPPARγ (Fig. 6E, F, *P* < 0.05). Therefore, the inhibition of miR-96-5p reversed the inflammatory and ECM degradation in the IL-1β-treated HNPCs via elevating the expression of PPARγ.

### Inhibition of miR-96-5p relieved IL-1β-induced HNPCs apoptosis by regulating PPARγ

**Fig. 7:**
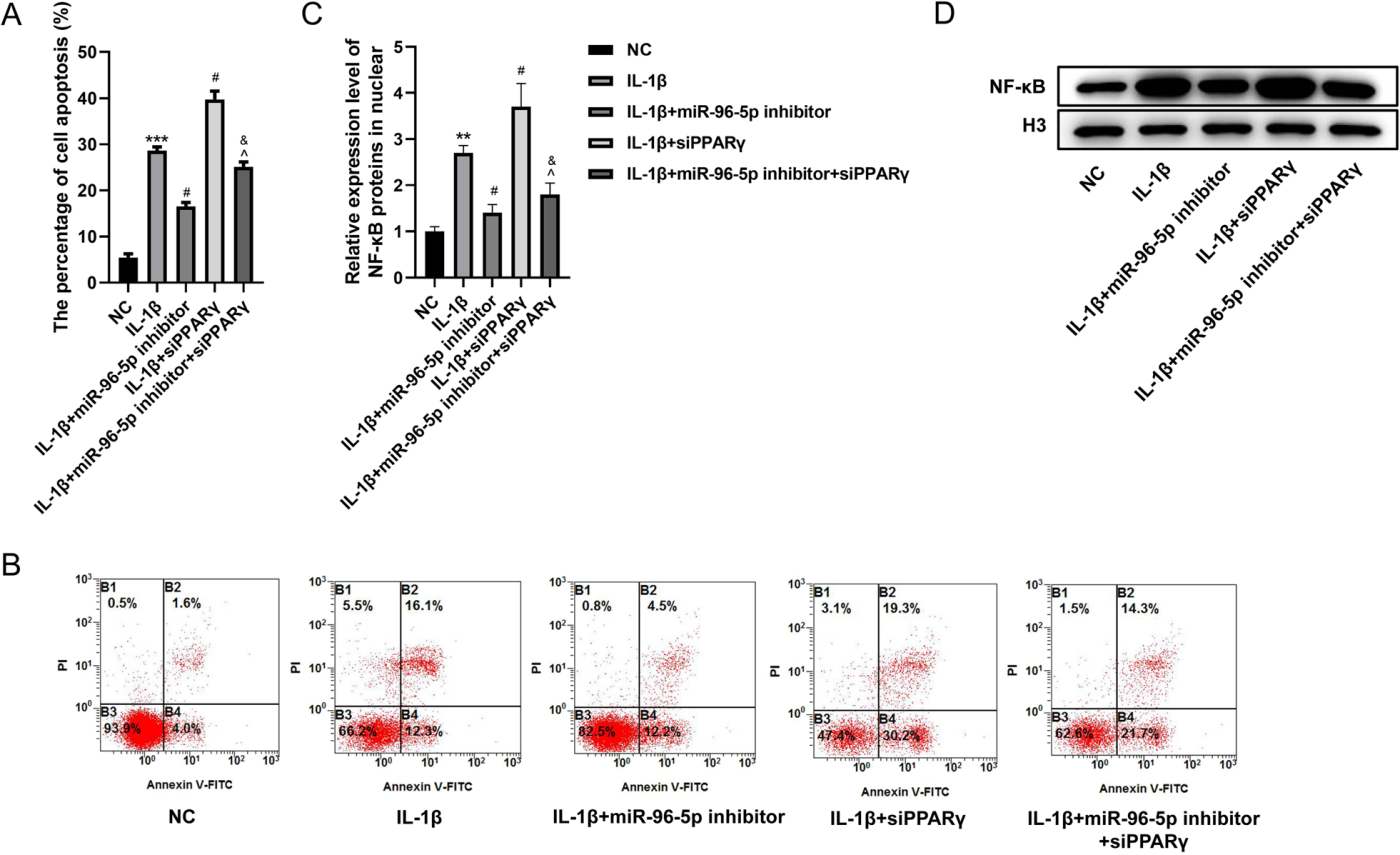
Inhibition of miR-96-5p relieved IL-1β-induced apoptosis of HNPCs by regulating PPARγ. HNPCs were treated with IL-1β and/or transfected with miR-96-5p inhibitor and/or short hairpin PPARγ (groups: NC, IL-1β, IL-1β + miR-96-5p inhibitor, IL-1β + siPPARγ, IL-1β + miR-96-5p inhibitor + siPPARγ). **A**, **B** Flow cytometry analysis with Annexin V-PI staining was performed to evaluate the percentage of apoptotic HNPCs. **C**, **D** Western blot assay was used to analyze the protein expression of NF-κB in the nucleus. (**P < 0.05, ***P < 0.01 *vs.* NC group; ^#^*P* < 0.05*vs.* IL-1β group; ^^^*P* < 0.05 *vs.* IL-1β + miR-96-5p inhibitor; ^&^*P* < 0.05 *vs.* IL-1β + siPPARγ). NC, normal control; IL-1β, interleukin-1β; PPARγ, peroxisome proliferator-activated receptor γ; siPPARγ, small interfering PPARγ; NF-κB, nuclear factor-kappaB; PI, propidium iodide; FITC, fluorescein isothiocyanate; HNPCs, human nucleus pulposus cells

The effect of miR-96-5p on the apoptosis of HNPCs in IDD via PPARγ was next explored and revealed the percentage of apoptosis decreased in the IDD cell model when miR-96-5p was inhibited (*P* < 0.05), while downregulation of PPARγ apparently restored this (Fig. 7A, B, *P* < 0.05). To investigate if miR-96-5p effected the PPARγ/NF-κB pathway, we then examined NF-κB level in the nucleus. As a result, the inhibition of miR-96-5p caused a much lower NF-κB level in the nucleus compared with the IDD cell model (*P* < 0.05), while siPPARγ partially reversed the inhibition of nuclear translocation of NF-κB caused by miR-96-5p inhibitor (Fig. 7C, D, *P* < 0.05). These results illustrated the inhibition of miR-96-5p relieved IL-1β-induced apoptosis of HNPCs by regulating PPARγ.

## Discussion

This study constructed an in vitro IDD model through IL-1β and showed that miR-96-5p was upregulated and the expression level of PPARγ was downregulated in IDD model. Mechanically, the inhibition of miR-96-5p could attenuate the IDD development by regulating the PPARγ/NF-κB signaling pathway, indicating miR-96-5p might be a promising target for the treatment of IDD.

IDD is an irreversible degenerative disease with an unclear pathophysiology and no effective treatment, resulting in a huge impact on the economy, public health, and the quality of life of patients [37]. Multiple intracellular signaling cascades are amenable to activation by inflammatory cytokines [38]. IL-1β has numerous pro-inflammatory properties reported to be correlated with the pathogenesis of IDD by inducing proteoglycan breakdown and inhibiting matrix biosynthesis by IVD cells [39, 40]. In this study, IL‑1β was utilized to stimulate HNPCs injury and was seen to reduce PPARγ level in HNPCs in a time- and dose-dependent manner. PPARγ level is effected by various inflammatory cytokines, and the administration of IL-17 and tumor necrosis factor-alpha (TNF-α) remarkably downregulated PPARγ expression in HNPCs [26]. One of the features of IDD is the secretion of inflammatory cytokines, and our results showed PPARγ could reduce IL-6 level in the medium of an IDD cell model.

The ECM is in a dynamic balance of constant anabolism and catabolism in normal IVD [41, 42]. However, during the IDD process, its metabolic imbalance and a progressive loss of Col II and aggrecan induce changes to the morphology and structure of IVD [43–47]. Pro-inflammatory cytokines can increase the levels of MMPs and ADAMTSs and promote the development of IDD [48–51]. Cao et al*.* showed the attenuation of inflammation and ECM accumulation in liver fibrosis was associated with the activation of PPARγ [52], while Poleni et al*.* found a PPARγ agonist significantly reduced transforming growth factor-beta1 (TGF-β1)-induced collagen deposition in rat chondrocytes [53]. Further, Mondragón et al*.* showed the controlled release and perfusion of the PPARγ inhibitor GW9662 promoted ECM deposition of human mesenchymal stem cells cultured on osteoinductive scaffolds [54]. Our study also found the upregulation of PPARγ protein and/or activation of PPARγ resulted in an increase in matrix components (Col II and aggrecan), and a decrease in MMPs (MMP3 and MMP13) and proteoglycanases (ADAMT-4 and ADAMT-5).

The apoptosis of HNPCs has an important influence on the progression of IDD [55–58], and leads to structural and mechanical instability of IVD [59, 60]. Ahsan et al*.* [61] found enhanced apoptosis in degenerative disc samples, and in the present work, a greater apoptosis rate of HNPCs in an IDD cell model was observed compared with the NC group. Further, upregulation and activation of PPARγ protein could alleviate apoptosis of HNPCs. Giampietro et al*.* [62] found the PPARγ agonist GL516 reduced the occurrence of apoptosis in rat astrocytes, while Kaundal et al*.* [63] found a PPAR agonist decreased the expression of apoptotic markers in rats with cerebral ischemia–reperfusion injury. Liu et al*.* [26] suggested the PPARγ agonist pioglitazone protected against IL-17-induced IVD inflammation and degeneration via regulation of the NF-κB signaling pathway, which is supported by the results of the present study. PPAR plays an important role in several orthopedic diseases. Pioglitazone was shown to partially protect animals from inflammatory induced-bone loss [64], and to inhibit osteoblast differentiation [65]. Agonists of PPARγ inhibited the development of cartilage lesions in part by suppressing inflammation [66].

PPARγ has been shown to suppress the inflammatory response by competitively repressing the NF-κB signaling pathway and terminating inflammation triggered by it. In this study, the level of PPARγ in degenerated disc tissues was obviously reduced compared to controls. Pioglitazone suppressed IDD by inhibiting NF-κB [26], and our results showed the upregulation and activation of PPARγ protein could inhibit NF-κB in an IDD cell model. PPARγ has also been reported to inhibit the NF-κB pathway in other disease, including acute lung injury induced by LPS [67]. Pioglitazone reduced the expression of NF-κB in mononuclear cells of peripheral blood in vitro [68], and a PPARγ agonist ( +)-(R,E)-6a1 inhibited the activation of NF-κB [69]. MiRNAs mediate cell proliferation, differentiation, apoptosis, and embryogenesis [59], and our study examined the effects of miR-96-5p and PPARγ/NF-κB on the development of IDD. MiR-96-5p has effects on diverse diseases, such as cancer [70–73], Parkinson’s disease [74], allergic rhinitis [75], neonatal sepsis [76], and wound healing [77], and has a vital impact on other orthopedic diseases. Suppression of syndecan-4 decreased cartilage degradation via miR-96-5p [78], while triptolide inactivated microglia via the miR-96/ikappaB kinase-beta (IKKβ)/NF-κB pathway and alleviated spinal cord injury [74]. Liu et al*.* [79] found miR-96 promoted osteogenic differentiation by targeting osterix, which may have been due to inactivation of the heparin-binding epidermal growth factor-like growth factor (HBEGF)-epidermal growth factor receptor (EGFR) pathway [80]. Nevertheless, the effect of miR-96-5p in IDD requires further exploration. In this research, miR-96-5p was shown to target PPARγ, and its inhibition could alleviate IDD by suppressing the NF-κB pathway. Regulation of the PPARγ/NF-κB pathway by miR-96-5p has been reported sparingly. MiR-96-5p reduced inflammatory responses via targeting nicotinamide phosphoribosyl transferase (NAMPT) to represses the NF-κB signaling pathway in RAW264.7 cells treated with LPS [76], while miR-96 targeted IKKβ to further regulate NF-κB, which is engaged in the inhibition of microglia activation and inflammatory factor secretion by raffinose methotrexate [81].

This study confirmed at the cellular level that miR-96-5p and NF-κB were increased in IDD, and PPARγ is decreased in IDD. It was clarified at the molecular and cellular levels that miR-96-5p could target the 3′-UTR region of PPARγ mRNA to inhibit PPARγ mRNA level, thereby regulating PPARγ protein level and NF-κB. In addition, this study also clarified at the cellular level that inhibiting miR-96-5p and activating PPARγ in HNPCs could alleviate the apoptosis and inflammation of HNPCs in IDD, and silencing PPARγ could reverse the alleviation of IDD by miR-96-5p inhibition. This further confirmed that miR-96-5p activated NF-κB by targeting inhibition of PPARγ, thereby promoting apoptosis and inflammation of HNPCs in IDD. Small interfering RNAs have been revealed to exhibit therapeutic purposes in various orthopedic diseases, such as human osteoporosis [82], human rheumatoid arthritis [83], and tendon healing [84]. This present study enriches the possible therapeutic targets of small interfering RNAs in IDD.

In addition, this study also has some shortcomings. Firstly, the correlation of the expression of miR-96-5p, PPARγ and NF-κB in IDD still need to be confirmed by further clinical samples. The molecular mechanism of PPARγ’s regulation of NF-κB protein also requires in-depth analysis. The role of miR-96-5p in alleviating IDD also deserves further in vivo experimental analysis.

## Conclusions

Taken together, this work examined inactivated PPARγ and the overexpression of miR-96-5p in IDD. The results showed the suppression of miR-96-5p could relieve IDD by activating the PPARγ/NF-κB signaling pathway, indicating miR-96-5p may be a promising target for IDD treatment. In the future, the discovery of drugs that target the suppression of miR-96-5p expression will provide new options for the treatment of IDD.

## Data Availability

The data used and analyzed during the current study are available from the corresponding authors on reasonable request.
